# Supplementary figures and images for: De Novo Assembly of the Polyhydroxybutyrate (PHB) Producer Azohydromonas lata Strain H1 Genome and Genomic Analysis of PHB Production Machinery
Source: Microorganisms. 2025 Jan 10;13(1):137. doi: 10.3390/microorganisms13010137 (PMC11767486; doi:10.3390/microorganisms13010137)

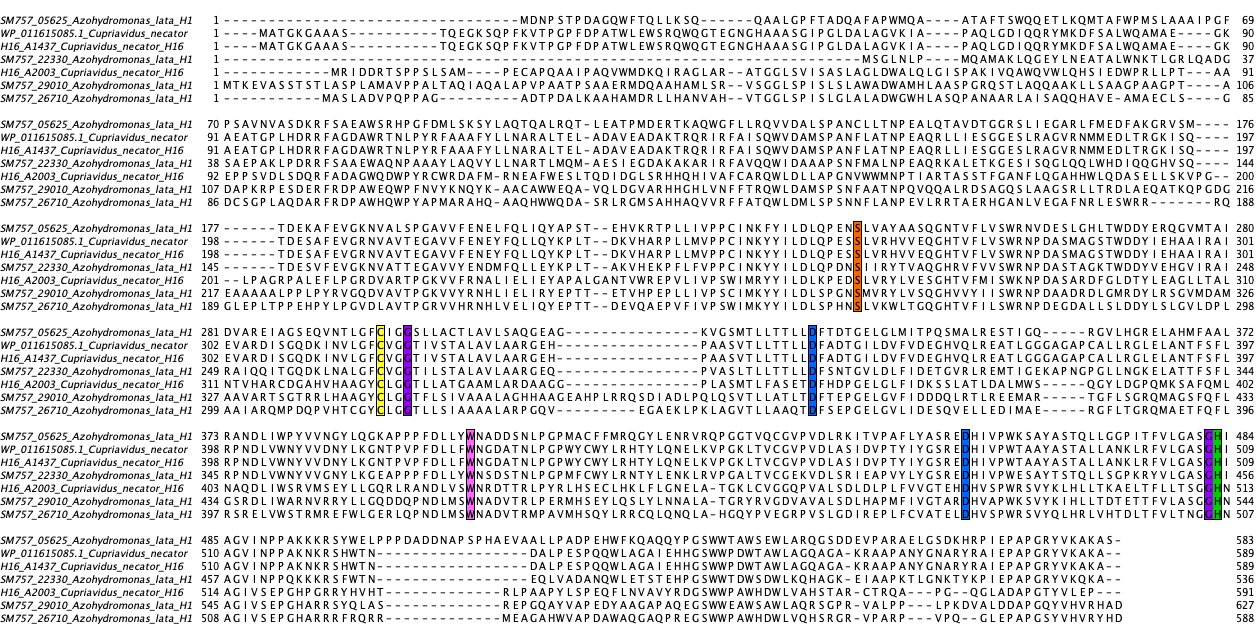

Supplement: Supplementary file 1 [file microorganisms-13-00137-s001.zip › Supplementary/FigureS1.png]
